# Evaluation of Magnetic Resonance Findings of Circumventricular Organs in Idiopathic Intracranial Hypertension Patients

**DOI:** 10.7759/cureus.31795

**Published:** 2022-11-22

**Authors:** Sule Nur Mraja, Ozlem Akdogan, Hamisi M Mraja, Ufuk Emre, Yeşim Karagöz

**Affiliations:** 1 Neurology, Duzce Ataturk State Hospital, Duzce, TUR; 2 Neurology, Health Sciences University, Istanbul Training and Research Hospital, Istanbul, TUR; 3 Scoliosis - Spine Center Istanbul, Istanbul Florence Nightingale Hospital, Istanbul, TUR; 4 Neurology, Istanbul Training and Research Hospital, Istanbul, TUR; 5 Radiology, Istanbul Education and Research Hospital, Istanbul, TUR

**Keywords:** cerebrospinal fluid, pineal gland, pituitary gland, magnetic resonance imaging, intracranial hypertension

## Abstract

Background: Idiopathic intracranial hypertension (IIH) is the increased pressure with normal cerebrospinal fluid (CSF) composition, not due to a secondary cause. However, the need for lumbar puncture, an invasive method in diagnosis, leads to research on noninvasive diagnostic methods. This study aims to examine the role of the size of the pituitary gland and the previously unevaluated pineal gland in radiological diagnosis in patients with IIH.

Materials and methods: The study retrospectively included 57 patients aged 18-80 years, who were followed up in our clinic with the diagnosis of IIH, and 52 control patients without central nervous system disease and cranial MR pathology. CSF pressure measurement values, CSF biochemistry, and cytology examinations were recorded as a result of lumbar puncture performed in the lateral decubitus position of all patients. In addition, the pineal gland and pituitary dimensions were measured by a neuroradiologist on cranial MR imaging of both groups.

Results: Pituitary gland height, anteroposterior (AP), and transverse dimensions were found to be significantly lower in the IIH patient group than in the control group (p<0.05). There were a significant reduction in pineal gland AP and height measurements in the IIH patient group compared to the control group. Still, we found no significant difference between the two groups in transverse measurements (p>0.05).

Conclusion: Our findings suggest that measurement of pituitary and pineal gland sizes in neuroimaging may be a guide as a noninvasive method in diagnosing and treating IIH.

## Introduction

Idiopathic intracranial hypertension (IIH) is an increase in intracranial pressure associated with a normal cerebrospinal fluid (CSF) composition, not due to a secondary etiology. Headache (75%-94%) and temporary vision loss (68%-72%) are the most common symptoms of IIH [1]. Although papilledema is a common and essential sign, pulsatile tinnitus and diplopia may also be seen. IIH most commonly occurs in young obese women of childbearing age. The leading causes of morbidity are severe headaches and vision loss if the disease is not diagnosed or delayed treatment [2]. The pathophysiology of IIH is still not fully understood. Hypothalamic-pituitary-adrenal axis disorders (Addison's disease, Cushing's syndrome, corticosteroid use or increased intracranial pressure in the female gender, pregnancy, polycystic ovary syndrome, obesity, and use of oral contraceptives suggest that there may be a hormonal relationship in the etiopathogenesis. It has been suggested that the neuroendocrine effect causes an increase in CSF production by the stimulation of mineralocorticoid receptors, which are abundant in choroid plexus epithelial cells [1].

The main neuroimaging findings that may accompany IIH are empty sella (70%), perioptic subarachnoid space enlargement (45%) and tortuosity (40%), flattening of the posterior sclera (80%), optic nerve papilla protrusion into the vitreous, and transverse sinus stenosis (90%) [2-7].

The pineal gland is among the circumventricular organs (CVOs), including the median eminence, subfornical organ, area postrema, subcommissural organ, organum vasculosum of the lamina terminalis, and posterior lobe of the pituitary gland. CVOs are highly vascularized structures located around the third and fourth ventricles and characterized by the absence of a blood-brain barrier. These specialized areas are communication points between blood, brain parenchyma, and CSF. The neurons and glial cells of CVOs form a unique repertoire of receptors and ion channels and receive many chemical signals from the bloodstream. These sensory CVOs, through their connections with the hypothalamus and brainstem, play a critical role in many homeostatic and nonhomeostatic functions such as sodium and water balance, cardiovascular regulation, energy metabolism, sleep-wake, body temperature, pain modulation, growth, lactation, reproduction, and immunomodulation [8]. In humans, roughly 80% of the pineal gland is composed of melatonin-producing pinealocytes, and the volume of the pineal gland (VPG) is proportional to melatonin levels in plasma, urine, or saliva [9]. Although the pineal gland is reported to develop after the first year of life entirely and does not change in size or weight later in life, recent studies have found that VPG could be changed by lifestyles such as coffee consumption or pathological conditions that may change melatonin production [10]. Melatonin affects many conditions and the development of diseases like obesity and related diseases such as sleep disorders, cardiovascular pathologies, and metabolic impairment. In many different neurological diseases such as dementia, CNS infections, and neurodegenerative diseases (such as Parkinson's disease), especially PG from the circumventricular organs has been evaluated and found to be associated with these diseases [10]. Given the effects of melatonin on these conditions and the association of melatonin with Pineal Gland volume, we may speculate that IIH patients have smaller Pineal Gland sizes than individuals without IIH, and Pineal sizes may predict the future risk of IIH and may facilitate the follow-up of the disease. 

Empty sella is one of the radiological findings of IIH. It is caused by the flattening of the pituitary in a sellar space filled with CSF. Studies have observed that findings such as pituitary morphology, cerebral venous structure, orbital structure, optic nerve extension, Meckel's caves, and especially sellar configuration have been evaluated, but there is no study examining the pineal gland in IIH patients [2]. The present study evaluated two neuroendocrine structures, the pituitary and pineal glands, by measuring their dimensions in three different planes on cranial MRIs. Both patients diagnosed with IIH and healthy control patients were compared. This study aimed to evaluate whether these measurements were significant in the diagnosis and follow-up of IIH.

## Materials and methods

**Figure 1:**
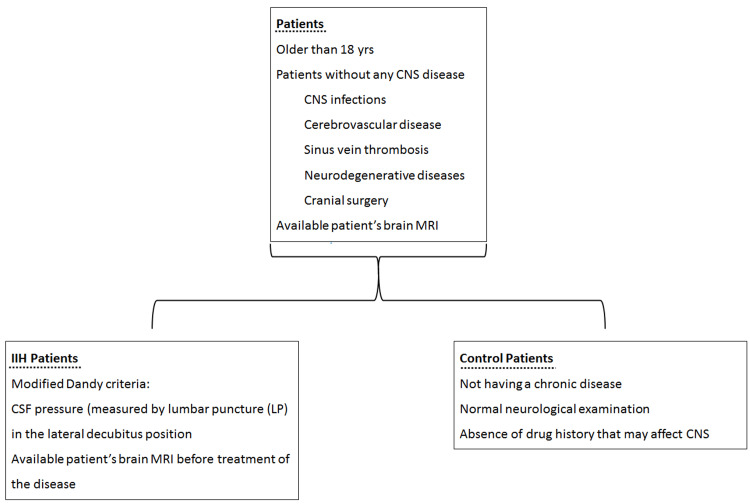
Inclusion criteria for IIH patient group and control group. CNS (central nervous system) MRI (Magnetic resonance imaging) CSF (cerebrospinal fluid) LP (Lumbar puncture)

Our study included 57 IIH patients and 52 control patients aged 18-80 years. In addition, patients diagnosed with IIH based on clinical examination, laboratory, and cranial MR findings in Istanbul Training and Research Hospital Neurology Clinic between January 1, 2016, and July 31, 2021, were evaluated retrospectively. This study was approved by the SBU Istanbul Training and Research Hospital Clinical Research Ethics Committee (2495, 21/08/2020). Figure 1 summarizes the inclusion process of IIH patients and healthy control patients for our study.

MRI procedure and measurements

**Figure 2:**
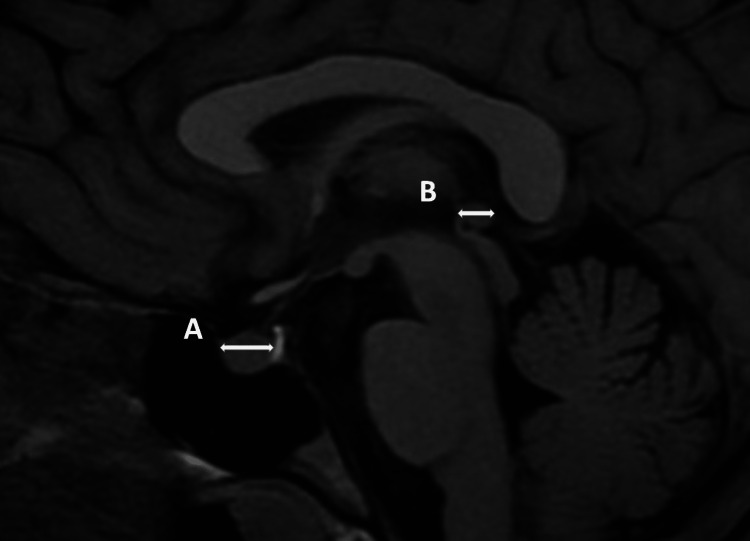
Sagittal MRI images demonstrate the height and anteroposterior length measurement of the pituitary gland (A) and the pineal gland (B).

**Figure 3:**
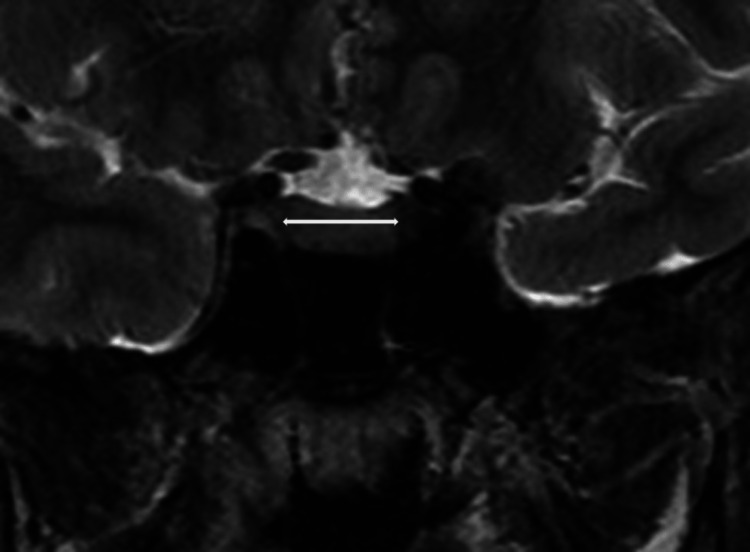
Coronal MRI images demonstrate the transverse length measurement of the pituitary gland.

**Figure 4:**
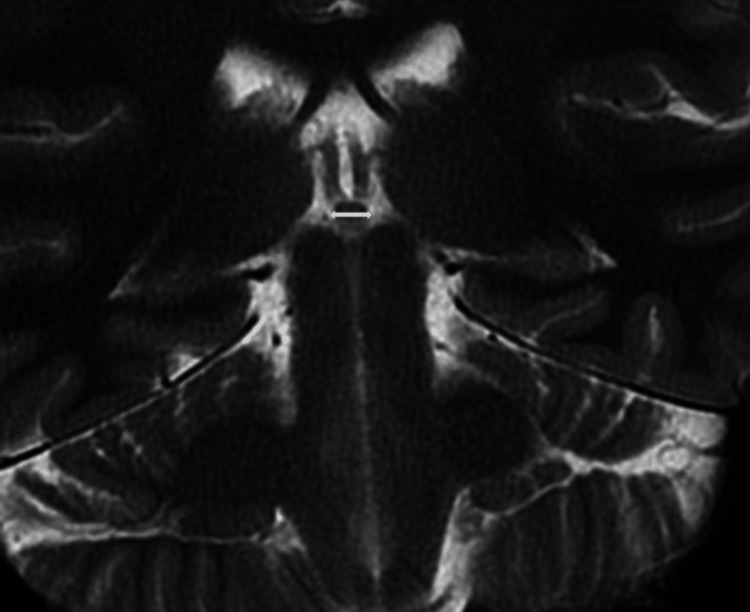
Coronal MRI images demonstrate the transverse length measurement of the pineal gland.

Cranial MR images were taken with a 1.5 T Magnetom Aera (Siemens, Erlangen, Germany) MR device with a 5.0 mm section thickness. Two experienced neuroradiologists evaluated the findings and were blinded to the patient's clinical data. The pituitary and pineal glands were evaluated for their height and anteroposterior (AP) length in the sagittal section and their transverse length in the coronal section (Figures 2-4).

Statistical analyses

The results of the analysis of all data were recorded as mean, standard deviation, median lowest, highest, frequency, and ratio values. Statistics of the findings were made with the SSPS 23 program. Data distributions were tested with the t-test, Mann-Whitney U test, and Kruskal-Wallis test (Nonparametric) for comparisons between the two groups, and the Tamhane test was used for post hoc analysis. Spearman Rank Correlation determined the degree of relationship between groups. p<0.05, p<0.01, and p<0.001 were considered statistically significant.

## Results

Demographic and clinical characteristics

In the study, 57 patients with IIH (55 females, two males) and 52 control patients (44 females, eight males) were included. The mean age of the IIH patient group was 41.2, and the control group was 35.2 years.

It was found that 55 of the patients (96%) presented with headache, 33 (57%) with visual symptoms in addition to headache, and 5 (8%) with headache and tinnitus in addition to visual symptoms. Two patients (3.5%) presented with only visual symptoms without headache. Of the 33 patients with visual symptoms at admission, 22 had blurred vision, three had double vision, six had intermittent vision loss, and two had flashing light.

Fifty IIH patients (87%) had a documented neurological examination. Only one IIH patient had an abnormal neurological examination, having a sixth cranial nerve palsy. Forty patients (70%) had recorded fundus examination, with papilledema identified in 37 (92%) at the initial neurological examination. Papilledema severity was determined according to the modified Frisén scale (MFS) as shown in Table 1 [11]. Optic atrophy was found in two patients. In addition, we observed that eight out of 10 patients whose visual field records were accessed had narrowed. Only two patients had a standard visual field.

**Table 1:** Papilledema grading using the modified Frisén scale (MFS)

Papilledema	n (%)
Normal	3 (7.5)
Grade 1	22 (55)
Grade 2	12 (30)
Grade 3	1 (2.5)
Grade 4	1 (2.5)
Grade 5	1 (2.5)

CSF opening pressure records of all patients were available, and the mean CSF pressure was found to be 35.81 cm-H_2_O (26 cm-H_2_O -63 cm-H_2_O). CSF biochemistry was normal in all patients included in the study.

Brain MRI evaluations

Cranial MR evaluations are shown in Table 2.

**Table 2:** MRI findings in the patients

Evaluated findings	n (%)
Empty sella	41 (71)
Partiel empty sella	19
Empty sella	22
Perineural fluid increase	15 (26.3)
Posterior globe flattening	2 (3.5)
Optic nerve tortiosity	10 (17.5)
Transverse sinus stenosis	10 (17.5)
Meckel cave expansion	3 (5)

Pituitary gland measurements

The mean values of the pituitary gland in the IIH patient group were measured as 8.6 mm in height, 13.05 mm in AP, and 19.8 mm in transverse. The control group measured these values as 6.1 mm in height, 5.92 mm in AP, and 9.7 mm in transverse, respectively. The mean values of the pineal gland in the IIH patient group were measured as 5.54 mm in height, 4.61 mm in AP, and 3.51 mm in transverse. The control group measured these pineal gland values as 3.47 mm in height, 7.05 mm in AP, and 5.1 mm in transverse, respectively.

**Figure 5:**
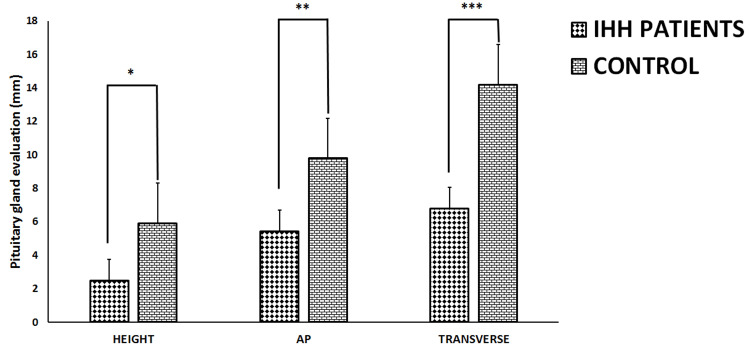
Pituitary gland evaluation of both the IIH patients and control groups. All data represented as Mean ± SD in millimeters (mm). *Height value of the pituitary gland was found to be significantly lower in the IIH patient group than in the control group (p<0.05). **AP and ***transverse values of the pituitary gland were significantly decreased in the IIH patient group compared to the control group (p<0.05). AP (anteroposterior) IIH (Idiopathic intracranial hypertension).

The height value of the pituitary gland was found to be significantly lower in the IIH patient group than in the control group (p<0.05). Similarly, AP and transverse values of the pituitary gland were significantly decreased in the IIH patient group compared to the control group (p<0.05) (Figure 5).

Pineal gland measurements

**Figure 6:**
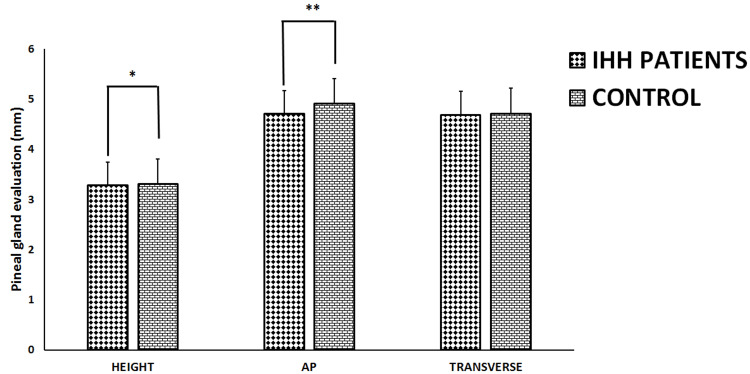
Pineal gland evaluation of both the IIH patients and control groups. All data represented as Mean ± SD millimeters (mm). *Height value was significantly lower in the IIH patient group than in the control group (p<0.05). Length value was significantly smaller in the IIH patient group compared to the control group (p<0.05). AP (anteroposterior) IIH (Idiopathic intracranial hypertension).

Pineal gland height was significantly lower in the IIH patient group than in the control group (p<0.05). Pineal gland AP length was significantly smaller in the IIH patient group compared to the control group (p<0.05). There was no difference between the patient and control groups regarding pineal gland transverse size (p>0.05) (Figure 6).

Correlational analyses

The correlation coefficient between the pituitary gland and pineal gland dimensions measured for the IIH patient and control groups was investigated. In addition, the pressure variable was included in these data in the IIH patient group. The rank correlation coefficient was used as the coefficient (Table 3).

**Table 3:** Correlation coefficients of the parameters measured in the IIH patient group. *A positive correlation between the pituitary gland height, pituitary gland AP and pituitary gland transverse values (p<0.05). **A moderate positive correlation between pineal gland height and AP measurements (p<0.05).

n=57	Pituitary Gland Height	Pituitary Gland AP	Pituitary Gland Transverse	Pineal Gland AP	Pineal Gland Height	Pineal Gland Transverse	Pressure
Pituitary Gland Height	1.00	0.883*	0.864*	-0.07	-0.25	-0.09	-0.04
Pituitary Gland AP	0.883*	1.00	0.909*	-0.08	-0.21	-0.15	0.02
Pituitary Gland Transverse	0.864*	0.909*	1.00	-0.08	-0.19	-0.20	-0.01
Pineal Gland AP	-0.07	-0.08	-0.08	1.00	0.430**	-0.02	0.14
Pineal Gland Height	-0.25	-0.21	-0.19	0.430**	1.00	0.13	0.07
Pineal Gland Transverse	-0.09	-0.15	-0.20	-0.02	0.13	1.00	0.00
Pressure	-0.04	0.02	-0.01	0.14	0.07	0.00	1.00

According to the IIH patient data in Table 3, it was observed that there was a high degree of positive correlation between the pituitary gland height, pituitary gland AP and pituitary gland transverse values (p<0.05). Furthermore, a moderate positive correlation was detected between pineal gland height and AP measurements (p<0.05). In addition, CSF pressure measured only in patients was not found to be associated with the pituitary gland and pineal gland sizes.

Correlation coefficients were also calculated for the control group. There was no correlation between the pituitary gland dimensions and the pineal gland dimensions in the control group. Likewise, there was no relationship between pineal gland dimensions among themselves and pituitary gland values among themselves in the control group.

There was no correlation between CSF opening pressure and pituitary gland and pineal gland values (p<0.05). Namely, an increase or decrease in CSF pressure was not associated with an increase or decrease in pituitary or pineal gland values. There was no correlation between the degree of papilledema and the size of the pituitary or pineal Gland (p>0.05).

When neuroradiological measurements were evaluated according to age (aged 18-40, 41-54, 55 and over) in the patient and control groups, there was a significant decrease in all dimensions of the pituitary dimensions (height, transverse length, AP length) with age (p<0.05). In contrast, no significant difference was found in the measurements for the pineal gland (p>0.05).

## Discussion

Our study aimed to evaluate the size of the circumventricular organs of the pituitary and pineal glands of patients with IIH and to determine their role in the diagnosis and follow-up of the disease. In our findings, pituitary and pineal gland height and transverse measurements were significantly lower in IIH patients. In addition, there was a significant difference in the pituitary gland that was increased in patients in the AP measurements, while no difference was found in the pineal gland measurements.

All pituitary dimensions (height, AP, transverse) were significantly lower in the IIH patient group than in the control group. The total estimated pituitary gland volume calculated using these measurements was also found to be significantly lower in the IIH patient group. All patients in both groups had their pituitary dimensions decrease proportionally to age. Pineal gland height and AP measurements were significantly lower in the IIH patient group. There was no significant difference in pineal gland sizes according to age in both groups. No correlation was found between CSF pressure values, all dimensions of the pituitary gland, and the height of the pineal gland in the patient group.

MRI, which is among the noninvasive methods in managing IIH, in which there are different mechanisms defined in its etiopathogenesis, but the exact causes cannot be determined yet, is the first preferred imaging method, especially for diagnosis and differential diagnosis. It determines the intracranial pathologies when the patient presents with symptoms of increased intracranial pressure by revealing the disease of intracranial structures in terms of parenchymal diseases or vascular pathologies and detection of obstructions in the CSF circulation. Also, MRIs are used in patients with recurrency symptoms after treatment or whenever new symptoms develop.

In previous MRI studies performed on IIH patients, structures such as cerebral veins, optic nerve sheath, Meckel caves, and especially the sella configuration were examined [2]. Pituitary deformity, optic nerve sheath enlargement, optic nerve tortuosity, optic disc edema, contrast enhancement, and posterior globe flattening were associated with IIH [12]. Empty sella turcica, a common radiological finding of the disease, was observed at 71% in our study, similar to the rate in the literature. Empty sella is associated with intrasellar herniation of the arachnoid mater and CSF, which flattens the pituitary gland and remodels the sella turcica [13]. In our study, according to the age of both groups, the pituitary diameters height, transverse and AP) decreased with age, as shown in previous studies [14].

The pituitary gland in the sella turcica is also affected in IIH patients. It has been observed that the height of the pituitary gland reaches its former dimensions after reducing the intracranial pressure [15-19]. Their findings suggest that the pituitary gland is not compressed but rather deformed and that the filling of the suprasellar cistern induces an empty sella. Our study found a significant decrease in the pituitary height in the IIH patient group compared to the controls. However, no explanatory findings could be obtained about whether there is a relationship between opening CSF pressure levels and the degree of empty sella in IIH patients [20].

The pineal gland is among the organ of homeostasis. It is associated with many pathological processes such as eating, sleep, and behavioral disorders by synthesizing melatonin. It has been previously studied and associated with Mild Cognitive Disorders progressing to Alzheimer's disease (AD), REM sleep disorders in AD, and Parkinson's disease [10,21]. Unlikely, none of the studies evaluated the pineal gland in IIH patients.

The previously studied results suggested that melatonin secretion was strongly correlated with pineal gland volume. Little is known about the mechanisms that reduce pineal gland volume. These mechanisms include chronic vascular inflammation, brain tissue hypoxia, intracranial pressure, and exposure to sunlight. Considering factors such as obesity, and hormonal disorders, which are modifiable in the development process of IIH, the etiopathogenesis of which has not been clarified, the results of the studies pointing out that these disorders may be related to melatonin levels, suggest that melatonin and therefore the diameters of the pineal gland may be a noninvasive guiding finding in the pathogenesis of IIH.

In our study, the height of the pineal gland, AP and transverse diameters was compared separately between the IIH patient and control groups, and height and AP size were significantly lower in the IIH patient group. Still, no significant difference was found between the two groups in terms of transverse diameters. However, an interesting correlation was observed between pituitary height and the estimated pineal volume obtained by multiplying the three longest diameters, suggesting a link between the two glands despite their different localization and functions. Furthermore, the fact that the mean pineal volume was low in the IIH patient group due to the decrease in pineal gland height and AP size suggests that melatonin levels affected by PG volume may be indirectly effective in the pathogenesis of the disease.

On the other hand, the pineal gland, which lacks the blood-brain barrier, may be directly sensitive to CSF content and CSF pressure. Hence, any changes in the CSF, such as increased intracranial pressure, may cause a decrease in the volume of the pineal gland. This decrease in pineal gland volume, which may occur due to possible mechanical effects, may cause decreased secretion of melatonin, which is responsible for regulating circadian rhythm and may cause IIH-triggering factors such as weight gain through sleep and nutritional disorders. Studies with large case series and measuring serum or CSF melatonin levels are needed to confirm all these possible hypotheses.

It has been shown that pineal gland volume and melatonin may play a role in energy homeostasis and pathophysiological mechanisms that contribute to the development of obesity. In addition, the data obtained from this study emphasized the idea that the replacement of melatonin deficiency could be a new strategy in the treatment of obesity [22]. Therefore, this connection between obesity and the pineal gland may also exist between the pineal gland and IIH since obesity also triggers the development of IIH. In addition, it may change our perspective on the treatment of IIH and perhaps bring up the use of melatonin therapy in these patients.

Due to cranial MRI section thickness, the median eminence from circumventricular organs could not be evaluated. For the same reason, we think that the volume estimation cannot be made as close to the truth as possible. In addition, a comparison between the treatments applied to the patients and the sizes of the pituitary and pineal glands could not be made.

The limitations of our study include the low number of IIH and control patients. Also, the inability to obtain details about the characteristics of the headache, which is the first complaint in most patients, is another limitation.

## Conclusions

Our study is the first to evaluate the pituitary and pineal gland volumes together in IIH patients and draw attention to the pineal gland and related pathogenetic mechanisms. We think that our study provides a different perspective on the pathogenesis and follow-up of IIH disease. Measurement of pituitary and pineal gland sizes in neuroimaging may be a guide as a noninvasive method in diagnosing and treating IIH. In addition, this evaluation has acknowledged the pituitary gland, which is affected by this disease, together with the pineal gland.
